# The Role of Metabolism in Heart Failure and Regeneration

**DOI:** 10.3389/fcvm.2021.702920

**Published:** 2021-07-16

**Authors:** Jiyoung Bae, Wyatt G. Paltzer, Ahmed I. Mahmoud

**Affiliations:** Department of Cell and Regenerative Biology, University of Wisconsin-Madison School of Medicine and Public Health, Madison, WI, United States

**Keywords:** heart regeneration, development, metabolism, heart failure, cell cycle

## Abstract

Heart failure is the leading cause of death worldwide. The inability of the adult mammalian heart to regenerate following injury results in the development of systolic heart failure. Thus, identifying novel approaches toward regenerating the adult heart has enormous therapeutic potential for adult heart failure. Mitochondrial metabolism is an essential homeostatic process for maintaining growth and survival. The emerging role of mitochondrial metabolism in controlling cell fate and function is beginning to be appreciated. Recent evidence suggests that metabolism controls biological processes including cell proliferation and differentiation, which has profound implications during development and regeneration. The regenerative potential of the mammalian heart is lost by the first week of postnatal development when cardiomyocytes exit the cell cycle and become terminally differentiated. This inability to regenerate following injury is correlated with the metabolic shift from glycolysis to fatty acid oxidation that occurs during heart maturation in the postnatal heart. Thus, understanding the mechanisms that regulate cardiac metabolism is key to unlocking metabolic interventions during development, disease, and regeneration. In this review, we will focus on the emerging role of metabolism in cardiac development and regeneration and discuss the potential of targeting metabolism for treatment of heart failure.

## Introduction

Heart failure is the leading cause of morbidity and mortality worldwide. In the United States alone, there are over 6,000,000 people with heart failure (1). This is largely due to the inability of the adult mammalian heart to replenish the lost myocardial tissue following injury, which results in the progressive weakening of the heart muscle and the development of heart failure (2). Current therapies are focused on preventing further remodeling of the remaining myocardial tissue. Heart transplantations are the only treatment in patients with severe heart failure (3). Due to the complexity and complications associated with heart transplants they are not always a suitable treatment; therefore, identifying novel therapeutic approaches to promote adult heart regeneration provides immense opportunities to advance heart failure therapy. Endogenous heart regeneration following injury has been demonstrated in some non-mammalian vertebrates (4, 5). Interestingly, neonatal mice are also capable of regenerating their heart tissue following injury, however this regenerative ability is lost within a few days following birth (6, 7). These models of endogenous regeneration provide us with a platform to elucidate the mechanisms that guide heart regeneration to reactivate these processes to promote adult heart regeneration.

**Figure 1:**
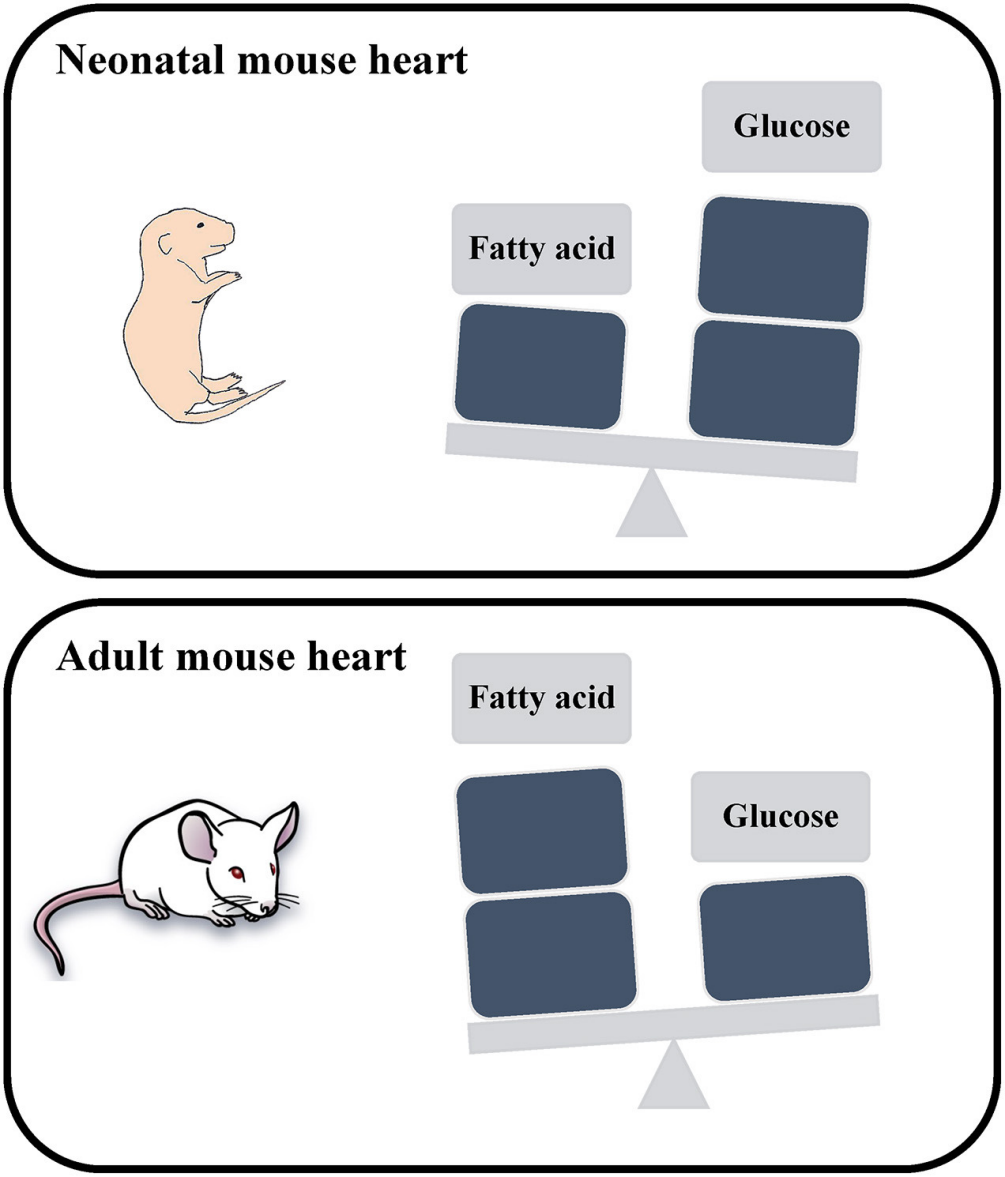
Schematic representation of the energy utilization in neonatal and adult mouse hearts. The neonatal mouse heart generates energy through glucose metabolism, while the adult mouse heart generates energy through fatty acid oxidation.

Embryonic and neonatal cardiomyocytes produce energy primarily *via* glycolysis, where postnatal maturation is accompanied with a metabolic switch to fatty acid oxidation to meet the energy demands of adult cardiomyocytes (8) (Figure 1). This metabolic switch contributes to the postnatal cardiomyocyte cell cycle exit and loss of the regenerative potential of the mammalian heart. This underscores the potential role of cardiac metabolism as a target to promote adult heart regeneration.

**Figure 2:**
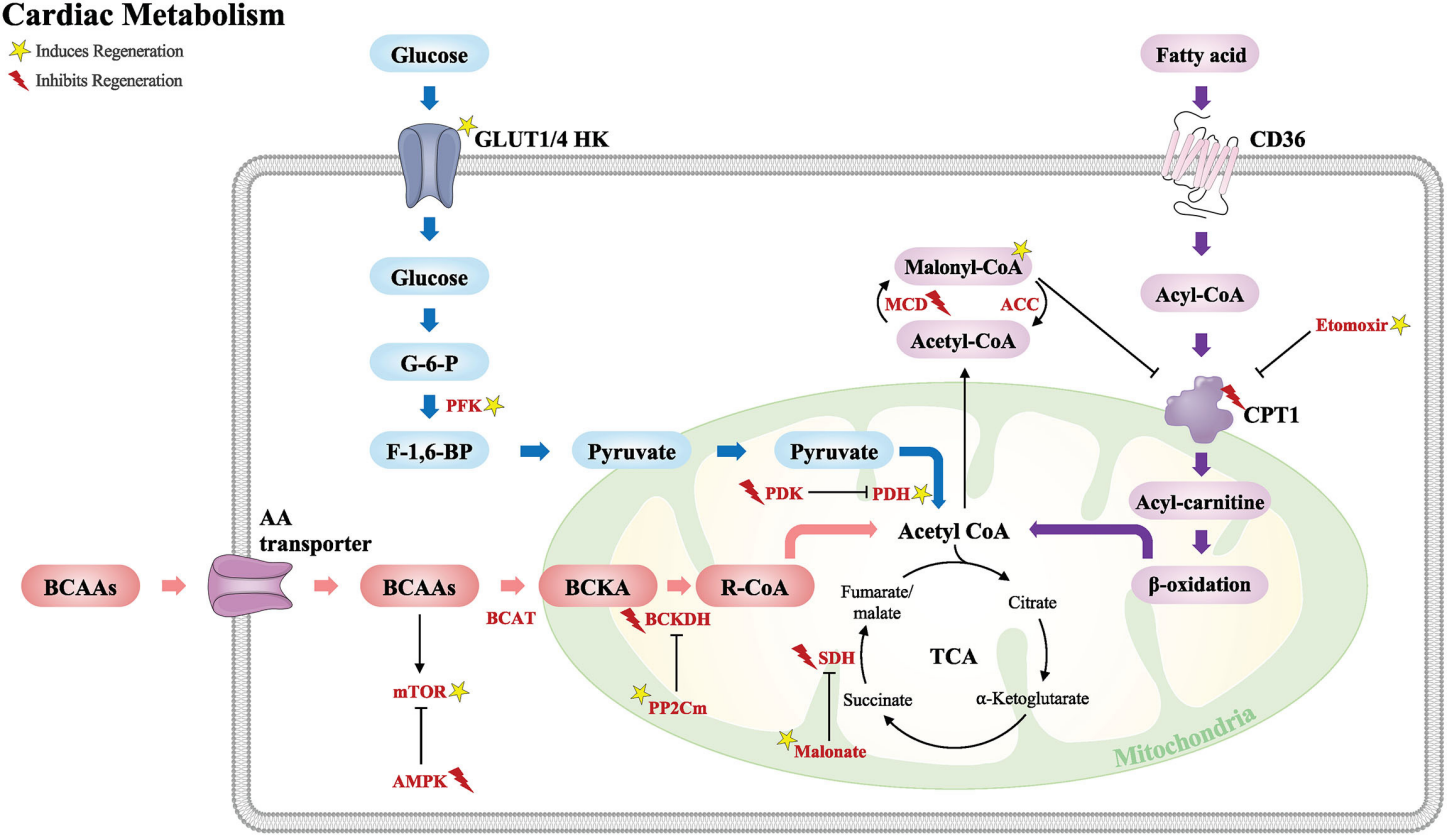
Schematic of the major metabolic pathways that modulate the cardiac regenerative response following injury. Glucose metabolism (blue), fatty acid metabolism (purple), and BCAA metabolism (red). Acetyl CoA from these major metabolic pathways is required for the TCA cycle. GLUT, glucose transporter type; HK, hexokinase; G-6-P, glucose-6-phosphate; F-1,6-BP, fructose-1,6-biphosphate; PFK, phosphofructokinase; PDK, pyruvate dehydrogenase kinase; PDH, pyruvate dehydrogenase; CD36, cluster of differentiation; CPT1, carnitine palmitoyltransferase; MCD, malonyl CoA dehydrogenase; ACC, acetyl CoA carboxylase; BCAAs, branched-chain amino acids; AA, amino acid; BCAT, branched-chain amino-transferase; BCKA, branched-chain alpha keto acids; BCKDH, branched-chain alpha-keto acid dehydrogenase; PP2Cm, protein phosphatase 2Cm; mTOR, the mechanistic target of rapamycin; AMPK, 5′ adenosine monophosphate-activated protein kinase; TCA, tricarboxylic acid cycle; SDH, succinate dehydrogenase. Yellow star induces regeneration and red lightning bolt inhibits regeneration.

In this review, we highlight major studies of cardiac metabolism including fatty acid oxidation, glucose, and amino acid metabolism (Figure 2). We also discuss key metabolic targets that may play an important role during cardiomyocyte development and regeneration and their potential as a therapeutic target for adult heart disease.

## Energy Metabolism and Heart Regeneration

The heart is the most energy-consuming tissue (per gram) in the human body (9), and energy production takes place in the mitochondria. The main function of the mitochondria is generating energy as adenosine triphosphate (ATP); thus, mitochondria play an essential role during development, cellular proliferation, and tissue regeneration, all of which are energy demanding processes (10–12).

Heart regenerative capacity differs by model organisms from vertebrates to human. Zebrafish and newts have a remarkable capability to regenerate their hearts after injury. Zebrafish heart regeneration is primarily derived from the proliferation of the pre-existing cardiomyocytes (13, 14). Following injury, cardiomyocyte dedifferentiation and proliferation are required to regenerate the heart following injury. Interestingly, single-cell transcriptional analysis of regenerating zebrafish hearts demonstrate that proliferating border zone cardiomyocytes undergo metabolic reprogramming to glycolysis from oxidative phosphorylation following cryoinjury (15). In contrast, glycolysis inhibitors including 2-deoxyglucose and lonidamine impair cardiomyocyte proliferation and heart regeneration (15). These results suggest that the glycolytic metabolic state mediates cardiomyocyte proliferation and regeneration following injury in zebrafish.

Like zebrafish, embryonic and neonatal mice can regenerate their heart after injury. Both zebrafish and neonatal mouse hearts have lower mitochondrial DNA copy numbers compared to postnatal and adult mice (16). This increase in cardiomyocyte mitochondrial number in the adult heart is due to the switch from glycolytic metabolism in neonatal cardiomyocytes to oxygen-dependent mitochondrial oxidative phosphorylation in adult cardiomyocytes (17). This metabolic switch and increase mitochondrial DNA results in a significant rise in reactive oxygen species (ROS) production from mitochondria which plays an essential role in regulating heart development and regeneration (16). This increase in ROS production contributes to postnatal cardiomyocyte cell cycle arrest. Interestingly, the increased sarcomere contraction in the postnatal heart promotes mitochondrial metabolism, which results in ROS production and DNA damage response activation *via* p53. As a result, inhibition of sarcomeres in cardiac troponin T knockout cardiomyocytes prevents cell cycle arrest and polyploidy resulting in increased cardiomyocyte proliferation (18). Furthermore, ROS scavengers such as N-acetyl cysteine (NAC) prolongs the postnatal window of cardiomyocyte proliferation and regeneration following ischemia reperfusion (I/R) injury (16).

Significant metabolic shifts occur in response to abnormal heart conditions. A healthy adult heart generates energy through fatty acid oxidation, however conditions such as pressure overload, hypertrophy, and ischemia results in a metabolic transition toward anaerobic glycolytic metabolism to be protect against damage (19). A recent study elegantly demonstrates the different metabolite utilization in human hearts by using arterio-venous metabolomics, which is a powerful tool to measure metabolite utilization in humans by measuring the metabolite intake and release in the blood from human hearts. Similar to mouse studies, healthy human hearts mostly uptake fatty acids as a fuel source while they only uptake limited amounts of glucose. Interestingly, the healthy heart releases amino acids, specifically essential amino acids. In contrast, the failing heart utilizes more ketones and lactate, but less fatty acids (20). These results are consistent with previous animal studies demonstrating that ketones and β-hydroxybutyrate are protective in the failing heart (21, 22). Collectively, these studies demonstrate that cardiac metabolism is dynamic and can switch to different states during development, disease, and regeneration.

## Fatty Acid Oxidation in the Heart

The heart requires high amounts of energy to maintain adult cardiac physiology (9). The adult human heart generates ATP *via* fatty acid oxidation (23–25). Fatty acids are oxidized through the tricarboxylic acid (TCA) cycle in the mitochondria, and the intermediate electrons from the TCA cycle flow through the electron transport chain (ETC) and produce a proton gradient to generate energy through ATP synthesis (26).

The first step for transporting long chain fatty acids from the cytosol into the mitochondria for initiating mitochondrial fatty acid oxidation occurs by carnitine palmitoyltransferase I (CPT1) in the outer mitochondrial membrane. CoA in acyl-CoA, which is derived from fatty acids, is converted to carnitine through CPT1. Thus, CPT1 is a key enzyme in regulating fatty acid oxidation. There are three tissue-specific isoforms of CPT1 that exist in mammalian tissues: CPT1A is expressed in the liver, lung, spleen, pancreas, and kidney; CPT1B is expressed in the heart, skeletal muscle, and adipose tissue; and CPT1C is expressed in the brain (27). Mitochondrial CPT1 activity is very low in the neonatal rat heart. Interestingly, CPT1 level is significantly increased in 7-day-old juvenile mice, which is the timepoint when the majority of mammalian cardiomyocytes have already exited the cell cycle (28). CPT1 expression is increased in adolescent (6 months) sheep hearts compared to fetus (105 days) hearts (29). Thus, CPT1 could be a key regulator of cardiomyocyte proliferation.

CPT1 inhibition reduces fatty acid oxidation due to the blockade of fatty acid transfer into the mitochondria. Inhibition of CPT1 by etomoxir promotes neonatal mouse cardiomyocyte proliferation (30). However, inhibition or activation of CPT1 does not induce cardiomyocyte proliferation in the adult mouse heart (31). Ventricular cardiomyocytes isolated from neonatal mice injected with the CPT1 inhibitor etomoxir show a reduction in fatty acid oxidation genes (30). These results demonstrate that disruption of fatty acid oxidation by inhibition of CPT1 extends neonatal cardiomyocyte proliferation and heart regeneration but is not sufficient to promote adult heart regeneration.

Another metabolite that regulates fatty acid oxidation *via* CPT1 inhibition is malonyl-CoA (32). Inhibition of malonyl-CoA decarboxylase (MCD), which is responsible for malonyl-CoA decarboxylation, results in increased malonyl-CoA levels which reduces fatty acid oxidation and increases glucose oxidation (33). As a consequence, short-term pharmacological inhibition of MCD increases malonyl-CoA levels in ischemic conditions resulting in improving cardiac function during ischemia/reperfusion (I/R) injury in the swine heart (34) and following myocardial infarction (MI) in the rat heart (35). Genetically MCD deficient mouse hearts show increased glucose oxidation and improved cardiac function following I/R injury (36). These results demonstrate that malonyl-CoA improves cardiac function following injury through CPT1 inhibition.

CPT1 is also regulated by peroxisome proliferator-activated receptors (PPARs), which are lipid receptors that play a critical role in regulating energy metabolism. There are three subtypes of PPAR: PPARα, PPARβ/δ, and PPARγ (37). PPARα, β/δ, γ gene expression levels are lower in the developing mouse heart compared to 14- and 28-day-old mouse hearts (38, 39). The levels of PPARs change during aging, as cardiac PPARα is significantly reduced in aged mice (40). PPARs play multiple roles in cardiac function in several disease states. It has been shown that expression of PPARα and CPT1 is notably reduced in adult mouse hearts following transverse aortic constriction (TAC) injury (41) as well as following I/R injury (42). However, activation of PPARα using the PPARα agonist GW7647 increased CPT1 gene expression which increased fatty acid oxidation and enhanced oxygen consumption rate in the presence of the fatty acid palmitate in isolated mouse cardiomyocytes (30).

However, the role of PPAR in cardiomyocyte proliferation and regeneration remains unclear. The PPARα agonist GW7647 does not promote cardiomyocyte proliferation and cardiac function following MI in adult mouse hearts (31). Furthermore, PPARα activation by agonist WY-14643 reduced cardiac function following I/R injury (42). Moreover, larger infarct size is observed in PPARα knockout mouse heart following I/R injury (43). In contrast, another study showed that PPARα transgenic mouse hearts showed improved cardiac function and reduced left ventricular dilation following TAC injury (41).

Another PPAR family receptor, PPARδ, has been shown to play a role during cardiac injury. The PPARδ ligand, GW501516, has been shown to inhibit cardiac fibroblast proliferation and transdifferentiation to myofibroblasts (44). Furthermore, inhibition of PPARδ reduced cardiomyocyte proliferation following injury in zebrafish hearts, whereas cardiomyocyte-specific PPARδ overexpression induced proliferation and reduced scar size following MI in mouse hearts (45).

Despite the important role of PPAR receptors in a variety of heart disease models, the exact role of these receptors in regulating cardiomyocyte proliferation and heart regeneration remains to be fully defined.

## Glucose Metabolism in Heart

Although the adult mammalian heart utilizes fatty acids as a main source of energy in the heart, glucose plays an important role as an energy source (46–49). Under healthy conditions the heart mostly uses fatty acids to produce energy, however, it will switch to glucose as an energy source during heart failure (50–52). Glucose metabolism is initiated by glucose uptake. In the heart, glucose enters cardiomyocytes *via* glucose transporters (GLUTs) which are expressed by various cell types. Among 14 members of the GLUT family (53), the most abundant GLUTs in the human heart are the insulin-sensitive glucose transporter GLUT4 (54, 55), and the insulin-independent glucose transporter GLUT1 (54, 56).

Under physiological conditions, GLUT1 is the main glucose transporter in embryonic and neonatal hearts, while GLUT4 is the primary glucose transporter in adult hearts (57, 58). In heart failure, GLUT4 expression is reduced while the levels of GLUT1 increase (59). This results in an increase in GLUT1-mediated glycolysis in heart failure, suggesting that GLUT1 plays an important role in cardiac protection during heart failure. GLUT1 expression is also increased in the heart under hypoxic conditions (60), which is mediated *via* hypoxia-inducible factor-1α (HIF-1α) (61). Cardiac-specific overexpression of GLUT1 results in increased glucose uptake and glycolysis in the mouse heart (62, 63), whereas cardiac-specific GLUT1 deletion reduces glucose uptake and glycolysis in isolated mouse cardiomyocytes following TAC injury (59). Interestingly, GLUT1 overexpression enhanced the regenerative response of neonatal mice following cryoinjury by increasing the levels of glucose metabolites (64). These results provide new evidence that increased GLUT1 expression promotes cardiomyocyte proliferation and heart regeneration through increased glucose metabolism.

Once glucose enters cardiomyocytes through GLUTs, glucose is phosphorylated and metabolized by key glycolytic enzymes such as hexokinase (HK) and phosphofructokinase (PFK) to form two pyruvate molecules (65). Pyruvate is then oxidized to acetyl CoA by pyruvate dehydrogenase (PDH), a key regulator in pyruvate metabolism (66), to enter the TCA cycle in the mitochondria. These glycolytic enzymes have been demonstrated to play a role in cardiac repair and regeneration following injury. In adult zebrafish, increased glycolysis has been shown to promote cardiomyocyte proliferation through increased cell cycle gene expression following injury (67). In addition, inhibition of glycolysis by 2-deoxyglucose reduced cardiomyocyte proliferation in the injured zebrafish heart (15). Thus, key components of glycolysis play an important role during cardiomyocyte proliferation and heart regeneration.

Hexokinase (HK) is the first enzyme of glycolysis that phosphorylates glucose to glucose-6-phosphate. Among the four distinct HK isozymes (HK 1, 2, 3, and 4) (68), HK-1 and -2 are expressed in the heart and regulate cardiac glucose metabolism (69, 70). Cardiac-specific HK-2 overexpression decreased cardiac hypertrophy in isoproterenol-induced mouse hearts and reduced cardiomyocyte size in neonatal rat ventricular cardiomyocytes (71). In addition, HK-2 overexpression reduced ROS accumulation which is upregulated during cardiac hypertrophy (71). In contrast, reduced HK-2 expression in HK-2^+/−^ mice results in increased cardiac dysfunction due to increase in cell death and fibrosis and reduction of angiogenesis following I//R injury (72). Whether HK plays a role during heart regeneration remains to be determined.

Another important enzyme that regulates glycolysis is phosphofructokinase (PFK) which has two isoforms: PFK-1 and PFK-2. PFK-2 regulates PFK-1 activity since PFK-2 regulates the synthesis of fructose-2,6-biphosphate, which activates PFK1 that promotes glycolysis. Thus, PFK-2 is a crucial enzyme that regulates glycolysis (65). PFK-2 is activated upon insulin stimulation which promotes glycolysis, where PFK-2 is reduced in the insulin-deficient streptozotocin-induced diabetic mice and high-fat diet-induced obese mice (73). Glycolysis and insulin sensitivity are decreased in cardiac-specific kinase-deficient PFK-2 mutant mouse hearts (74, 75). As a result, glycolysis is not increased in cardiac-specific kinase-deficient PFK-2 mice in contrast to wild type mice following TAC surgery (75). On the other hand, overexpression of kinase-active PFK-2 enhances contractility in hypoxic mouse cardiomyocytes (76). Thus, PFK-2 regulates glycolysis and may play a role in cardiac protection following injury.

A key glycolysis enzyme is pyruvate dehydrogenase kinase (PDK). There are four PDK isoforms (PDK 1, 2, 3, and 4). PDKs expression is significantly increased during heart development and is further increased in the adult heart (58). PDKs expression is also increased in the infarct zone following cardiac cryoinjury in zebrafish (67). Among the PDK isoforms, cardiac PDK4 is the most significantly upregulated enzyme in 7-day-old mice, where the majority of mammalian cardiomyocytes exit the cell cycle (58). PDKs play in a role in glycolysis *via* inhibition of pyruvate dehydrogenase (PDH), which is a limiting step in glucose oxidation. PDK inhibition by dichloroacetate induces PDH activation which promotes cardiac function following KCl-induced cardiac arrest (77). A recent study demonstrated that cardiac-specific deletion of PDK4 promotes adult cardiomyocyte proliferation and heart regeneration following adult MI (78). In summary, PDK plays an important role in glycolysis *via* inhibition of PDH activity, suggesting that PDKs may be an important therapeutic target to increase glycolysis and promote cardiac repair and regeneration.

Pyruvate kinase muscle isoenzyme 2 (PKM2), a rate-limiting enzyme in the final step of glycolysis, is expressed in embryonic and neonatal mouse hearts; however, it is significantly reduced beyond postnatal day 7 when cardiomyocytes exit the cell cycle (79). Interestingly, overexpression of PKM2 in cardiomyocytes promotes cell cycle and glucose-6-phosphate dehydrogenase expression (79). Cardiomyocyte-specific PKM2 expression by modified RNA (modRNA) promotes adult cardiomyocyte proliferation and cardiac regeneration following adult MI (79). Conversely, loss of PKM2 reduces cardiomyocyte proliferation following injury in zebrafish hearts (67). Moreover, cardiomyocyte-specific deletion of PKM2 impairs heart development as they exhibit smaller heart size and low levels of cardiomyocyte proliferation (79).

Taken together, these studies demonstrate that glycolysis plays an important role in regulating cardiomyocyte proliferation and heart regeneration following injury. Thus, targeting glucose metabolism is a promising approach to promote adult heart regeneration.

## Amino Acid Metabolism in the Heart

Amino acids are key molecules for cell growth and survival. Amino acids are used as the building blocks for protein synthesis as well as inhibiting proteolysis (80). In addition, amino acids serve as precursors to key metabolites (81). Remarkably, amino acids can act as a signaling molecule, such as leucine, which stimulates muscle protein synthesis *via* the mechanistic target of rapamycin (mTOR) signaling pathway (82–84). The levels of cellular amino acids fluctuate throughout development, increasing in postnatal stages until reaching peak levels around P9 and then decreasing into adult stages suggesting a dynamic role for amino acids during development and maturation (85).

A recent study demonstrated that circulating arterial amino acid levels are reduced in patients with heart failure in comparison to healthy patients (86). Decreasing levels of arterial amino acids correlated with reduced heart function, demonstrating the potential use for arterial amino acid levels as a biomarker of heart failure (86). To understand if this reduction of circulating arterial amino acids was the heart reducing its energy consumption of amino acids a recent study aimed to quantify fuel consumption of the failing and non-failing human heart (20). This study demonstrated that energy consumption of amino acids was unchanged between the non-failing and failing hearts (20), suggesting that the role amino acids play in heart failure is not tied to their function as an energy source.

To further understand the role of amino acid metabolism in heart failure, a main focus was placed on a subset of amino acids, the branched chain amino acids (BCAAs), which are utilized differently than the other amino acids. BCAAs consist of leucine, isoleucine, and valine (87). BCAAs account for nearly 5% of total carbon used within the heart, and they also act as regulatory components for other metabolic processes (20, 88). BCAA catabolism has been shown to play a role in heart failure. This is seen in both humans and rodents where all components in BCAA catabolism have altered expression levels in heart failure (87). A study using a mouse model deficient in protein phosphatase 2Cm (PP2Cm), which is a critical component in the conversion of branch chain ketone acids to acyl-CoA derivatives *via* the branched-chain alpha-keto acid dehydrogenase complex (BCKDH), demonstrated that the knockout mice have a higher susceptibility to heart failure in response to pressure overload stress (87). This was due to the higher levels of BCAAs in the PP2Cm deficient mice, which reduced glucose breakdown *via* direct inhibition of pyruvate dehydrogenase (89).

The mechanistic target of rapamycin (mTOR) signaling pathway has been demonstrated to play an important role during heart development and growth (90, 91). Interestingly, BCAAs stimulate mTOR activation which promotes metabolic reprogramming to glycolysis from fatty acid oxidation through HIF-1α (92). In contrast, inhibition of mTOR promotes human iPSC-derived cardiomyocyte maturation and impairs zebrafish heart regeneration following injury (93, 94). mTOR is also inhibited by 5′ adenosine monophosphate-activated protein kinase (AMPK) through tuberous sclerosis complex 2 (TSC2) (95). Pharmacological activation of AMPK by metformin inhibits mTOR pathway activation following TAC injury (96). In addition, AMPK activation by AICAR promotes human iPSC-derived cardiomyocyte maturation (97). Thus, downstream pathways of BCAAs including mTOR and AMPK can regulate cardiomyocyte proliferation and regeneration.

Conversely, stimulating BCAA catabolism can be protective against heart injury and failure. BCAA catabolism can be activated by inhibition of the branched chain ketoacid dehydrogenase kinase (BCKDK), which results in BCKDH activation (87). BCKDK inhibition increased BCAA catabolism, which increased cardiac function following TAC compared to controls (98). In addition, adenoviral overexpression of PP2Cm in infarcted diabetic mice resulted in a significantly smaller scar size compared to controls (99). These studies demonstrate that enhanced BCAA catabolism can be protective against cardiac injury.

This relationship between BCAA catabolism and heart failure demonstrate that amino acid metabolism plays a role in heart disease and repair. Future studies to dissect the role of amino acids in the heart will establish their role as an important therapeutic target in cardiovascular disease.

## TCA Cycle Metabolites in the Heart

The metabolic switch from glycolysis in neonatal mice to fatty acid oxidation in adult cardiomyocytes is accompanied by a significant increase in mitochondrial number and high levels of ROS production (16). This increase in ROS levels in the postnatal heart induces cardiomyocyte DNA damage, which contributes to cardiomyocyte cell cycle exit in the adult mammalian heart (16). Thus, elucidating the role of mitochondrial metabolites in regulating this metabolic switch is critical to identify metabolic targets to promote adult heart regeneration.

Succinate dehydrogenase (SDH), also known as mitochondrial complex II, is an important enzyme in regulating cell cycle and metabolic reprogramming in cancer because SDH plays a role in both the TCA cycle and the electron transport chain (100). Metabolic reprogramming has been recognized as a hallmark of various cancers due to the unique metabolic signature of cancer (101). In the presence of oxygen, pyruvate is converted to acetyl-CoA which enters the mitochondrial TCA cycle. However, in the absence of oxygen very limited oxidative phosphorylation takes place, instead lactate production increases aerobic glycolysis (101). Interestingly, pyruvate is mostly converted to lactate in cancer cells regardless of the oxygen levels. This metabolic switch promotes cancer cell survival and cell proliferation (100–102).

Recent studies demonstrated that reverse activity of SDH during ischemia results in succinate accumulation (103, 104). The accumulated succinate is then rapidly oxidized following reperfusion and results in a burst of ROS production *via* reverse activity of the mitochondrial complex I (105). These studies suggest that ROS production due to reverse activity of SDH and succinate accumulation is a hallmark of I/R injury (105). Interestingly, SDH inhibition reduces infarct size during ischemia in Langendorff-perfused mouse hearts (106). Furthermore, the SDH competitive inhibitor malonate reduces infarct size during I/R injury in pig hearts (107). These results demonstrate that SDH inhibition during I/R injury blocks the SDH-mediated succinate accumulation, thus protecting the heart against the redox insult during I/R injury. Interestingly, a recent study demonstrated that succinate accumulation in ischemia/reperfusion is not due to the reverse activity of SDH, but rather due to canonical TCA cycle activity (108). Thus, although succinate accumulation during ischemia is conserved across vertebrates, the proposed mechanism of succinate accumulation remains to be further understood.

SDH knockdown induces cell proliferation and migration in human hepatocellular carcinoma cell lines and leads to a metabolic shift to glycolysis as demonstrated by increased level of glycolytic enzymes (109). Interestingly, a recent study demonstrated that metabolic reprogramming to glycolysis promotes cardiomyocyte proliferation and heart regeneration following injury in zebrafish (15). Remarkably, SDH inhibition by malonate promotes adult cardiomyocyte proliferation, revascularization, and heart regeneration following adult myocardial infarction (110). In contrast to the cardioprotective role of malonate during I/R injury in mouse and pig hearts (105, 107); malonate did not protect against infarction post-MI but rather promoted regeneration following infarction (110). Furthermore, SDH inhibition by malonate following adult MI was accompanied by increased succinate levels as a consequence of TCA cycle inhibition, which is distinct from the cardioprotective role of malonate that prevents succinate accumulation during I/R injury (105, 110). Interestingly, metabolic profiling of the adult heart demonstrated an increase in glucose metabolism and a decrease in TCA cycle metabolism following SDH inhibition by malonate, consistent with a metabolic reprogramming from oxidative phosphorylation to glycolysis in the adult heart. These results demonstrate that SDH inhibition by malonate promotes adult heart regeneration *via* metabolic reprogramming (110).

Collectively, these studies demonstrate an important role for mitochondrial metabolites in regulating the cardiac metabolic state, and targeting metabolism has an important therapeutic potential to promote adult heart regeneration.

## Discussion

The role of the complex metabolic interactions in the heart and their potential to promote cardiac repair and regeneration are beginning to be appreciated. The shift in metabolism from glycolysis to fatty acid oxidation after birth coincides with the loss of regenerative potential in the neonatal mouse heart. The studies that are highlighted throughout this review demonstrate that manipulation of metabolic pathways is an area of immense potential for identifying new therapeutics to treat heart diseases (Table 1). Targeting these metabolic pathways can promote or inhibit regeneration depending upon the specific component that is modulated (Figure 2).

**Table 1:** Summary of recent studies demonstrating a central role for metabolism in heart failure and regeneration.

**Metabolism**	**Target gene**	**Function**	**Application**	**Results**	**References**
Fatty acid oxidation	Carnitine palmitoyltransferase 1 (CPT1)	Induces fatty acid oxidation	CPT1 inhibition	Increased proliferation of isolated neonatal cardiomyocytes	(30)
				Reduced in fatty acid oxidation gene expression	
				No change in adult mouse cardiomyocyte proliferation	(31)
	Malonyl-CoA decarboxylase (MCD)	Reduces fatty acid oxidation	MCD inhibition	Increased malonyl-CoA levels in ischemic swine heart	(33, 34)
				Improved cardiac function following rat heart myocardial infarction (MI)	(35)
				Increased glucose oxidation in MCD deficient mouse heart	(36)
				Improved cardiac function in ischemic MCD deficient mouse heart	
	Peroxisome proliferator-activated receptor (PPAR) α	Induces fatty acid oxidation	PPARα activation	Increased CPT1 gene expression and oxygen consumption rate in the presence of the fatty acid palmitate in isolated mouse cardiomyocytes	(30)
				No change in adult cardiomyocyte proliferation and cardiac function following MI	(31)
				Cardiac function decreased following I/R injury	(42)
	PPARδ	Induces fatty acid oxidation	PPARδ activation	Decreased cardiac fibroblast proliferation and myofibroblast transdifferentiation	(44)
				Reduced cardiomyocyte proliferation and increased scar size following MI in mouse heart	(45)
			PPARδ inhibition	Reduced cardiomyocyte proliferation following cardiac injury in zebrafish	(45)
Glucose metabolism	GLUT1	Increases glucose uptake	GLUT1 overexpression	Increased glucose uptake and glycolysis in the mouse heart	(62, 63)
				Increased regenerative response and glucose metabolites in neonatal mouse heart following cryoinjury	(64)
		Decreases glucose uptake	GLUT1 inhibition	Reduced glucose uptake and glycolysis in isolated mouse cardiomyocytes following TAC injury	(59)
	Hexokinase (HK) 2	Increases glycolysis	HK-2 overexpression	Decreased cardiac hypertrophy in isoproterenol-induced mouse hearts	(71)
				Reduced cardiomyocyte size in neonatal rat ventricular cardiomyocytes	
				Reduced ROS accumulation	
		Decreases glycolysis	HK-2 inhibition	Increased cardiac dysfunction and cell death and fibrosis	(72)
				Decreased angiogenesis following I/R injury	
	Phosphofructokinase (PFK) 2	Increases glycolysis	PFK-2 inhibition	Reduced glycolysis and insulin sensitivity in mice	(74, 75)
			PFK-2 overexpression	Increased contractility in hypoxic mouse cardiomyocytes	(76)
	Pyruvate dehydrogenase kinase (PDK)	Increases glycolysis	PDK inhibition	Increased cardiac function following KCI-induced cardiac arrest	(77)
			PDK-4 inhibition	Promoted mouse cardiomyocyte proliferation and heart regeneration following adult MI	(78)
	Pyruvate kinase muscle isoenzyme 2 (PKM2)	Increases glycolysis	PKM2 overexpression	Increased cardiomyocyte proliferation and cardiac regeneration following adult MI	(79)
			PKM2 inhibition	Reduced cardiomyocyte proliferation following injury in zebrafish hearts	(67)
				Impaired heart development and reduced cardiomyocyte proliferation	(79)
Amino acid metabolism	Protein Phosphatase 2cm (PP2 cm)/Protein Phosphatase 1 k (PPM1K)	Reduced BCAA oxidation	PP2cm inhibition	Increased BCAA and BCKA levels	(87)
				Reduced cardiac function and increased heart failure	(87, 89)
				Decrease in glucose uptake and utilization	(89)
		Increased BCAA oxidation	PP2cm overexpression	Decreased DNA damage and cell death, leading to a smaller scar size post-MI	(99)
	BCKDK	Increased BCAA oxidation	BCKDK inhibition	Decreased free BCAAs, leading to improved heart function post-TAC	(98)
TCA cycle metabolism	Succinate dehydrogenase (SDH)	Reduced succinate accumulation	SDH inhibition	Reduced infarct size during ischemia in I/R mouse hearts	(106)
				Reduced infarct size during I/R injury in pig hearts	(107)
				Induced glucose metabolism in adult mouse hearts	(110)
				Promoted adult cardiomyocyte proliferation, revascularization, and heart regeneration following MI	(110)

Manipulating metabolic components in ways that can stimulate glucose metabolism has been implicated in promoting regeneration, as this shifts the heart's metabolic landscape closer to the metabolic state of the regenerative neonatal heart. This was demonstrated with deletion of PDK4, overexpression of PP2cm, as well as SDH inhibition *via* malonate, which promoted regeneration by inducing glucose metabolism *via* modulating their respective metabolic pathways (78, 99, 110).

In contrast, increased fatty acid oxidation has been demonstrated to reduce the cardiac regenerative response following injury. Inducing fatty acid oxidation *via* treatment with the PPARα agonist WY-14643 results in reduced cardiac function after injury (42). Similarly, inhibition of glycolysis exacerbates cardiac injury, as demonstrated by reduced HK-2 expression (72) and PP2cm deletion (87).

The dynamic role of glycolysis and fatty acid oxidation following injury demonstrates a central role for cardiac metabolism during regeneration. Although multiple key components have already been identified that can be targeted therapeutically, these metabolic pathways play an important role in cardiac homeostasis. Thus, elucidating the mechanisms of these pathways during homeostasis, disease, and regeneration is an essential step prior to targeting these pathways for therapeutic development. For example, targeting succinate dehydrogenase post-MI promoted adult heart regeneration, yet the mechanisms by which succinate dehydrogenase inhibition promotes regeneration needs to be fully understood prior to clinical use (110). Furthermore, harnessing the potential of known pharmacological agents that have been demonstrated to target these metabolic pathways needs to be explored as candidates to induce adult heart regeneration.

Elucidating the role of cardiac metabolism in health and disease will provide us with novel avenues with significant therapeutic potential that could aid in promoting heart repair and regeneration. Advancements in this area of research will provide a better understanding of heart disease and regeneration.

## Author Contributions

JB and AM contributed to conception and design of the manuscript. JB, WP, and AM wrote the manuscript. All authors contributed to manuscript revision, read, and approved the submitted manuscript.

## Conflict of Interest

The authors declare that the research was conducted in the absence of any commercial or financial relationships that could be construed as a potential conflict of interest.
